# Different Cytokine and Chemokine Expression Patterns in Malignant Compared to Those in Nonmalignant Renal Cells

**DOI:** 10.1155/2017/7190546

**Published:** 2017-07-09

**Authors:** Nadine Gelbrich, Hannes Ahrend, Anne Kaul, Lars-Ove Brandenburg, Uwe Zimmermann, Alexander Mustea, Martin Burchardt, Denis Gümbel, Matthias B. Stope

**Affiliations:** ^1^Department of Urology, University Medicine Greifswald, Greifswald, Germany; ^2^Department of Trauma, Reconstructive Surgery and Rehabilitation Medicine, University Medicine Greifswald, Greifswald, Germany; ^3^Department of Gynaecology and Obstetrics, University Medicine Greifswald, Greifswald, Germany; ^4^Department of Anatomy and Cell Biology, RWTH Aachen University, Aachen, Germany; ^5^Department of Trauma and Orthopaedic Surgery, BG Klinikum Unfallkrankenhaus Berlin gGmbH, Berlin, Germany

## Abstract

**Objective:**

Cytokines and chemokines are widely involved in cancer cell progression and thus represent promising candidate factors for new biomarkers.

**Methods:**

Four renal cell cancer (RCC) cell lines (Caki-1, 786-O, RCC4, and A498) and a nonmalignant renal cell line (RC-124) were examined with respect to their proliferation. The cytokine and chemokine expression pattern was examined by a DNA array (Human Cytokines & Chemokines RT^2^ Profiler PCR Array; Qiagen, Hilden, Germany), and expression profiles were compared.

**Results:**

Caki-1 and 786-O cells exhibited significantly increased proliferation rates, whereas RCC4 and A498 cells demonstrated attenuated proliferation, compared to nonmalignant RC-124 cells. Expression analysis revealed 52 cytokines and chemokines primarily involved in proliferation and inflammation and differentially expressed not only in malignant and nonmalignant renal cells but also in the four RCC cell lines.

**Conclusion:**

This is the first study examining the expression of 84 cytokines and chemokines in four RCC cell lines compared to that in a nonmalignant renal cell line. VEGFA, NODAL, and BMP6 correlated with RCC cell line proliferation and, thus, may represent putative clinical biomarkers for RCC progression as well as for RCC diagnosis and prognosis.

## 1. Introduction

Renal cell cancer (RCC) represents the deadliest neoplasm of the urinary tract, due to the fact that a majority of patients are diagnosed at a very advanced stage [1, 2]. Tumor progression is based on various molecular mechanisms, and thus, further examinations of RCC tumor biology are necessary in order to understand the progression and, furthermore, to identify novel biomarkers for diagnosis and prognosis of RCC [1, 3–5].

Among other mechanisms, cytokines and chemokines are suspected to play a crucial role in proliferation and progression of various malignancies. For instance, CCL11 controls cancer cell growth and invasion in ovarian cancer and CCL21 as well as CCR7 represent pivotal regulators of bladder cancer progression [6, 7]. Moreover, IL17, as a representative of interleukins, is associated with lung and colorectal cancer progression and predicts poor prognosis in breast cancer [8–10]. Even though some studies suggested an impact in RCC, the role of cytokines and chemokines in RCC progression is poorly understood [1, 11, 12]. From a clinical point of view, cytokine and chemokine secretion into the blood stream makes them very suitable as noninvasive clinical markers.

In the study presented here, we used an RCC model system consisting of four malignant RCC cell lines compared to a nonmalignant renal cell line. After classification of the RCC cells in cell lines of high and low cell growth rates, a transcriptional profiling specific for 84 cytokines and chemokines (Table 1) was carried out and compared to the corresponding cell growth properties. The aim of this analysis was to identify cell growth-associated cytokines and chemokines by comparison of malignant and nonmalignant expression patterns, particularly with regard to the identification of putative biomarkers for RCC progression.

## 2. Materials and Methods

### 2.1. Cell Culture

RCC cell lines Caki-1, 786-O, A498 (Cell Lines Service, Eppelheim, Germany), and RCC4 (Sigma-Aldrich, München, Germany) and the nonmalignant renal cell line RC-124 (Cell Lines Service) were used. Caki-1 and A498 cells were cultivated in a minimum essential medium (MEM) supplemented with 79.6 mg/l nonessential amino acids, 2 mM L-glutamine, 1 mM sodium pyruvate, 1% penicillin/streptomycin (P/S), and 10% FBS (PAN Biotech, Aidenbach, Germany). 786-O cells were propagated in RPMI 1640 medium containing 2 mM L-glutamine, 1% P/S, and 10% FBS (PAN Biotech); RCC4 cells were cultivated in Dulbecco's Modified Eagle Medium (DMEM) with 1 mM sodium pyruvate, 1% P/S, and 10% FBS (PAN Biotech); and RC-124 cells were cultivated in McCoy's 5a medium supplemented with 2 mM L-glutamine, 1% P/S, and 10% FBS (PAN Biotech). All cells were propagated in a humidified atmosphere (37°C, 5% CO_2_).

### 2.2. Proliferation Assay

Proliferation of cells was examined by cell counting (CASY Cell Analyzer, Roche Applied Science, Mannheim, Germany). Adherent cells, detached by trypsin/ethylenediaminetetraacetic acid (EDTA) treatment, were suspended in CASYton (Roche Applied Science) as 1 : 100 dilution. Measurement was performed using a capillary of 150 *μ*m in diameter and cell line-specific gate settings to discriminate between living cells, dead cells, and cellular debris (Caki-1: 8.57 *μ*m/15.4 *μ*m, 786-O: 6.9 *μ*m/14.7 *μ*m, RCC4: 8.5 *μ*m/15.75 *μ*m, A-498: 7.2 *μ*m/15.75 *μ*m, and RC-124: 6.5 *μ*m/12.75 *μ*m).

### 2.3. Quantitative DNA Array

RCC cells and nonmalignant RC-124 cells were grown on a 6-well cell culture plate to 80% confluency and total RNA was prepared (peqGOLD TriFast reagent, Peqlab Biotechnology, Erlangen, Germany). Analysis of 84 human cytokines and chemokines (Table 1) was performed using the Human Cytokines & Chemokines RT^2^ Profiler PCR Array (Qiagen, Hilden, Germany) according to the supplier's instructions (http://www.sabiosciences.com). Briefly, 0.5 *μ*g of total RNA was reversely transcribed and polymerase chain reaction (PCR) was done on a CFX96 Real-Time System (Bio-Rad, Munich, Germany) and analyzed with the CFX Manager software (Bio-Rad).

### 2.4. Enzyme-Linked Immunosorbent Assay (ELISA)

IL6, IL8, IL11, IL15, and VEGF concentrations in cell culture supernatant of RCC cells and nonmalignant RC-124 cells were determined by use of target-specific DuoSet ELISA kits with a Substrate Reagent Pack containing stabilized hydrogen peroxide and tetramethylbenzidine (all R&D, Minneapolis, MN, USA) according to the supplier's instructions. Cells were sedimented (1.3 × g, 5 min), the supernatant was incubated with capture antibodies (overnight, 4°C), and soluble antigens were determined applying the BMG FLUOstar OPTIMA Microplate Reader with OPTIMA software 2.10 (BMG Labtech, Offenbach, Germany). Each sample was analyzed in duplicates.

### 2.5. Statistics

For data evaluation, the graphics and statistics software Graph Pad Prism V5.01 (GraphPad Software, La Jolla, CA, USA) was used. Principal component analysis (PCA) was performed using Multibase 2015 software (NumericalDynamics.Com, Tokyo, Japan).

## 3. Results

**Figure 1:**
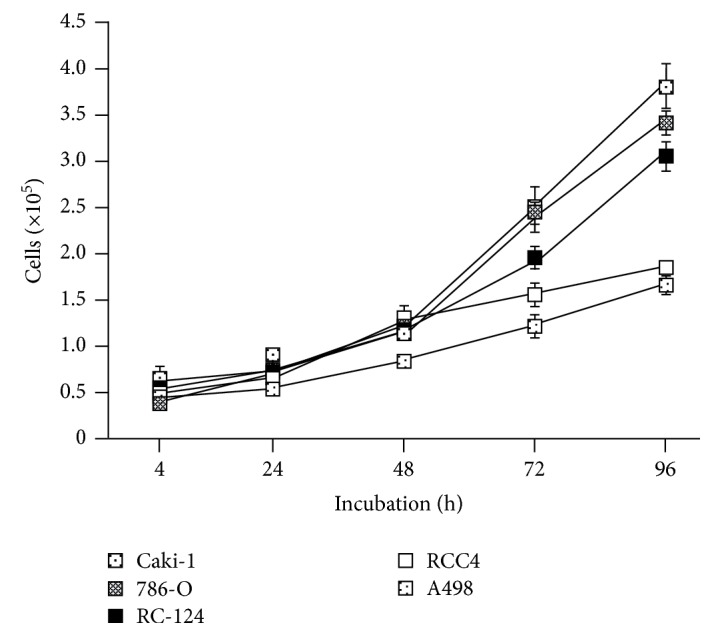
Cellular growth of the RCC cell lines Caki-1, 786-O, RCC4, and A498 as well as the nonmalignant renal cell line RC-124. Cellular growth was examined at indicated time points utilizing a CASY Cell Counter and Analyzer Model TT (Roche Applied Science). Results are expressed as the mean ± SD of cell count.

In contrast to the elevated proliferation rates of Caki-1 and 786-O cells compared to those of nonmalignant RC-124 cells, both, RCC4 and A498 cells, showed a markedly reduced cell growth (Figure 1). These differences in proliferation prompted us to investigate whether cytokines and chemokines may control the individual growth properties of the RCC cell lines.

**Figure 2:**
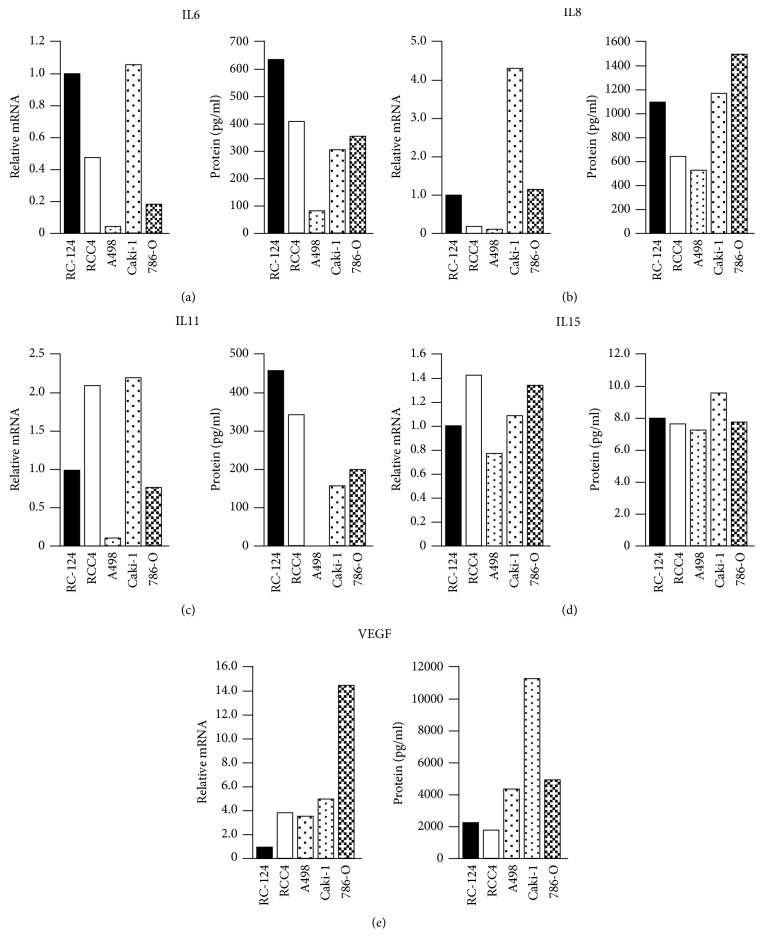
Comparison of mRNA levels and protein levels of selected cytokines. Steady-state levels of intracellular mRNA levels and extracellular protein levels of IL6 (a), IL8 (b), IL11 (c), IL15 (d), and VEGF (e) were assessed by quantitative DNA array (Human Cytokines & Chemokines RT^2^ Profiler PCR Array; Qiagen) and enzyme-linked immunosorbent assay (ELISA) analysis, respectively. mRNA amounts are expressed as relative mRNA levels normalized to nonmalignant RC-124 cells (RC-124 mRNA = 1.0). Protein levels are expressed as pg protein/ml cell culture supernatant.

Performing the RT^2^ Profiler PCR Array Human Cytokines & Chemokines, the expression of 84 target mRNAs (Table 1) was measured. From these 84 factors, which have been examined at the intracellular mRNA level, the 5 cytokines IL6, IL8, IL11, IL15, and VEGF have been exemplarily chosen for verification at the extracellular protein level via ELISA. mRNA concentrations of IL6, IL8, IL11, IL15, and VEGF appeared to correspond to the levels of secreted proteins of the RCC cells and the nonmalignant renal cell line (Figure 2). The transcript pattern of IL6, IL8, and IL15 was very comparable to the levels of secreted proteins in all cell lines. However, there were differences in Caki-1 cells with respect to the low level of secreted IL11 protein and the high level of secreted VEGF protein compared to that of intracellular mRNA. Exactly opposite results were found with respect to 786-O cells. Nevertheless, comparison of cytokines' mRNA and protein levels revealed a clear correlation indicating mRNA arrays' applicability for expression analyses of cytokines and chemokines.

**Figure 3:**
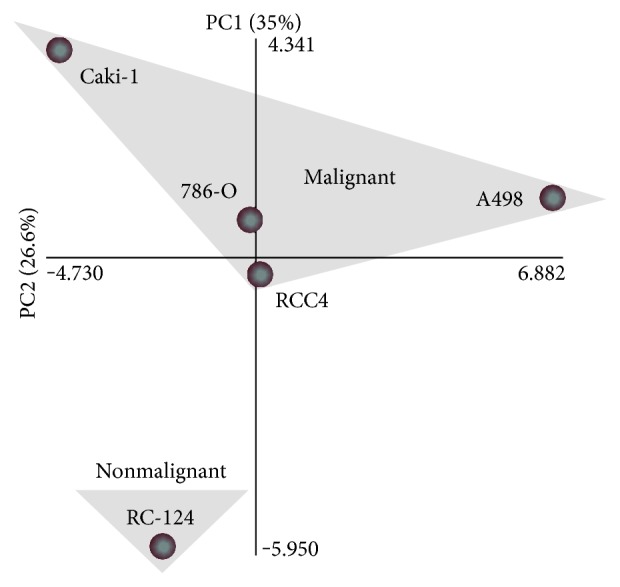
Principal component analysis (PCA) of the cytokine and chemokine expression. The RCC cell lines Caki-1, 786-O, RCC4, and A498 as well as the nonmalignant renal cell line RC-124 were plotted in a two-dimensional coordinate plane defined by the principal components 1 (PC1) and 2 (PC2) analyzing the expression data of all cytokines and chemokines measured by the DNA array (Human Cytokines & Chemokines RT^2^ Profiler PCR Array; Qiagen). Clustering of the malignant cells is indicated by a grey triangle.

Furthermore, a global normalized PCA was performed and used as quality check. PCA was carried out on the concentrations of all of the detected cytokines and chemokines in all of the five cell lines (Figure 3) dividing cancer and noncancer cells (PC1) and defining individual characteristics of all cell lines (PC2). As expected, the five cell lines used in this study were divided correctly into two major groups corresponding to malignant *versus* nonmalignant physiology.

Performing the Human Cytokines & Chemokines RT^2^ Profiler PCR Array, the expression of various cytokines and chemokines for each of the cell lines was analyzed compared to the nonmalignant cell line RC-124. The following 32 cytokines and chemokines were not expressed: ADIPQ, BMP7, CCL1, CCL17, CCL18, CCL19, CCL22, CCL24, CCL3, CCL8, CD40LG, CXCL13, CXCL9, FASLG, IFNG, IL10, IL12B, IL13, IL16, IL17A, IL17F, IL21, IL22, IL24, IL3, IL4, IL5, IL9, LTA, THPO, TNFSF11, and XCL1.

**Figure 4:**
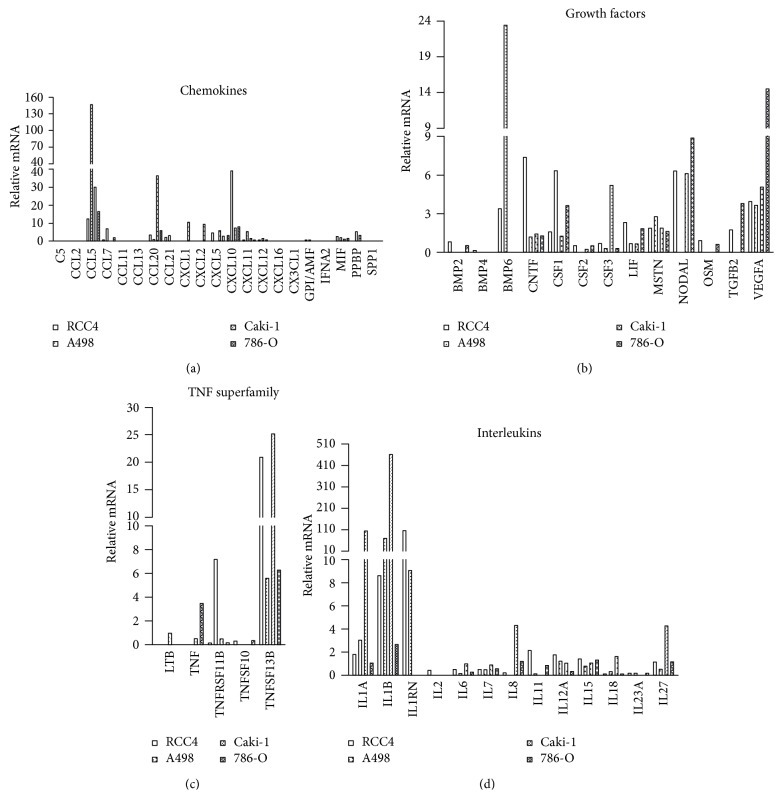
Expression profile of cytokines and chemokines in RCC cell lines. Expression data of cytokines and chemokines in the RCC cell lines Caki-1, 786-O, RCC4, and A498 as well as in the nonmalignant renal cell line RC-124 were measured performing a DNA array (Human Cytokines & Chemokines RT^2^ Profiler PCR Array; Qiagen) and expressed as relative mRNA normalized to nonmalignant RC-124 cells (RC-124 = 1.0). Cytokines and chemokines were classified into the four groups of chemokines (a), growth factors (b), TNF superfamily members (c), and interleukins (d).

52 factors were modulated compared to RC-124 cells (Figure 4). The group of chemokines (Figure 4(a)) was dominated by the overexpression of CCL20, CXCL10, and foremost CCL5 with more than a 100-fold overexpression. Most striking was the strong upregulation of these three chemokines in A498 cells, even compared to the other RCC cell lines. Within the family of growth factors, a more balanced distribution pattern of the factors was demonstrated (Figure 4(b)). However, only CNTF, CSF1, CSF3, LIF, MSTN, and VEGFA were detectable in all of the four malignant cell lines.

Five members of the TNF superfamily were detected (Figure 4(c)); however, only TNFSF13B was strongly overexpressed in all of the four malignant cell lines, dominated by an upto 25-fold overexpression in Caki-1 and RCC4 cells. Considering the family of interleukins (Figure 4(d)), a total of 13 different factors were detected with an upregulated expression of the interleukins IL1A, IL1B, and IL1RN in each of the 4 malignant cell lines.

## 4. Discussion

The process of tumorigenesis is driven by several mechanisms including an increased proliferation, angiogenesis, and immunomodulation [13]. Cytokines and chemokines, in general, are primarily regulators of immune defense. CCL5, in particular, supports leukocyte diapedesis by integrin induction [14, 15]. Interestingly, CCL5 expression in all four RCC cell lines was highly upregulated. Since tumorigenesis is associated with inflammatory response, elevated CCL5 secretion by RCC cells may be part of cancer-related inflammation. Corresponding to this observation is the finding that the expression levels of proinflammatory interleukins IL1A and IL1B are increased in all of the four RCC cell lines. Interestingly, overexpression of the anti-inflammatory IL1RN [16] could only be observed in the lowly proliferative RCC4 and A498 cells. This could mean that IL1RN may interfere with antiproliferative pathways or that anti-inflammatory processes generally attenuate cell growth.

Within the TNF superfamily, TNFSF13B is significantly overexpressed in all four RCC cell lines. Looking at the large differences in cellular growth of highly proliferative Caki-1 and 786-O and lowly proliferative RCC4 and A498 cell lines, however, classification of TNFSF13B as a proliferative factor in RCC, as has been done in previous studies [17], could not be clearly confirmed.

As expected, expression levels of proliferative cytokines, for example, CSF-1, CSF-3, NODAL, and VEGFA, correlated with the cell growth rates of the highly proliferative (Caki-1, 786-O) and the lowly proliferative (RCC4, A498) RCC cells. This observation has been confirmed by several studies. CSF-1 and VEGFA expression is associated with RCC progression and bad prognosis for patient survival [11, 18, 19]. Furthermore, elevated levels of NODAL, a member of the TGF*β* superfamily, confer enhanced tumorigenicity [20–23]. Expectedly, strongly overexpressed proliferative factors, for example, VEGFA and NODAL, may represent novel prognostic markers for RCC.

Vice versa, the expression of BMP6 was significantly increased in the lowly proliferative cell lines RCC4 and A498, whereas an expression in highly proliferative Caki-1 and 786-O cells was not detectable. Since BMP6 is considered to be a tumor suppressor in breast cancer [24], upregulation of BMP6 in RCC cells may contribute to attenuated cell growth of RCC4 and A498 cells compared to that of the nonmalignant cell line RC-124.

Taken together, there are a number of cytokines and chemokines which are differentially expressed not only in malignant and nonmalignant renal cells but also in the four RCC cell lines. These results may reflect heterogeneity among RCC patients as well as among RCC subtypes. In addition, this may suggest a functional substitution of members of the cytokine/chemokine system due to an activation of alternative factors involved in cytokine/chemokine signalling. Notably, two proliferative factors (VEGFA, NODAL) and a tumor suppressive factor (BMP6) have been found which may significantly contribute to the malignant phenotype of RCC and thus may be suitable as novel marker proteins in RCC diagnosis and therapy.

## Figures and Tables

**Table 1:** Human Cytokines & Chemokines RT^2^ Profiler PCR Array (Qiagen). Abbreviation (symbol) and full name/function (description) of the 84 cytokines and chemokines analyzed by the PCR array. The column “detectable” indicates whether the factor was detected (+) or not detected (−) within this study.

Number	Symbol	Description	Detectable
Chemokines			
1	C5	Complement component 5	+
2	CCL1	Chemokine (C-C motif) ligand 1	−
3	CCL2	Chemokine (C-C motif) ligand 2	+
4	CCL3	Chemokine (C-C motif) ligand 3	−
5	CCL5	Chemokine (C-C motif) ligand 5	+
6	CCL7	Chemokine (C-C motif) ligand 7	+
7	CCL8	Chemokine (C-C motif) ligand 8	−
8	CCL11	Chemokine (C-C motif) ligand 11	+
9	CCL13	Chemokine (C-C motif) ligand 13	+
10	CCL17	Chemokine (C-C motif) ligand 17	−
11	CCL18	Chemokine (C-C motif) ligand 18	−
12	CCL19	Chemokine (C-C motif) ligand 19	−
13	CCL20	Chemokine (C-C motif) ligand 20	+
14	CCL21	Chemokine (C-C motif) ligand 21	+
15	CCL22	Chemokine (C-C motif) ligand 22	−
16	CCL24	Chemokine (C-C motif) ligand 24	−
17	CXCL1	Chemokine (C-X-C motif) ligand 1	+
18	CXCL2	Chemokine (C-X-C motif) ligand 2	+
19	CXCL5	Chemokine (C-X-C motif) ligand 5	+
20	CXCL9	Chemokine (C-X-C motif) ligand 9	−
21	CXCL10	Chemokine (C-X-C motif) ligand 10	+
22	CXCL11	Chemokine (C-X-C motif) ligand 11	+
23	CXCL12	Chemokine (C-X-C motif) ligand 12	+
24	CXCL13	Chemokine (C-X-C motif) ligand 13	−
25	CXCL16	Chemokine (C-X-C motif) ligand 16	+
26	CX3CL1	Chemokine (C-X3-C motif) ligand 1	+
27	GPI/AMF	Glucose-6-phosphate isomerase	+
28	IFNA2	Interferon, alpha 2	+
29	MIF	Macrophage migration inhibitory factor	+
30	PPBP	Proplatelet basic protein (chemokine (C-X-C motif) ligand 7)	+
31	SPP1	Secreted phosphoprotein 1	+
32	XCL1	Chemokine (C motif) ligand 1	−
Growth factors			
33	BMP2	Bone morphogenetic protein 2	+
34	BMP4	Bone morphogenetic protein 4	+
35	BMP6	Bone morphogenetic protein 6	+
36	BMP7	Bone morphogenetic protein 7	−
37	CNTF	Ciliary neurotrophic factor	+
38	CSF1	Colony stimulating factor 1 (macrophage)	+
39	CSF2	Colony stimulating factor 2 (granulocyte-macrophage)	+
40	CSF3	Colony stimulating factor 3 (macrophage)	+
41	LIF	Leukemia inhibitory factor (cholinergic differentiation factor)	+
42	MSTN	Myostatin	+
43	NODAL	Nodal homolog (mouse)	+
44	OSM	Oncostatin M	+
45	TGFB2	Transforming growth factor, beta 2	+
46	THPO	Thrombopoietin	−
47	VEGFA	Vascular endothelial growth factor A	+
TNF superfamily			
48	CD40LG	CD40 ligand	−
49	FASLG	Fas ligand (TNF superfamily, member 6)	−
50	LTA	Lymphotoxin alpha (TNF superfamily, member 1)	−
51	LTB	Lymphotoxin beta (TNF superfamily, member 3)	+
52	TNF	Tumor necrosis factor	+
53	TNFRSF11B	Tumor necrosis factor receptor superfamily, member 11b	+
54	TNFSF10	Tumor necrosis factor (ligand) superfamily, member 10	+
55	TNFSF11	Tumor necrosis factor (ligand) superfamily, member 11	−
56	TNFSF13B	Tumor necrosis factor (ligand) superfamily, member 13b	+
Interleukins			
57	IL1A	Interleukin 1, alpha	+
58	IL1B	Interleukin 1, beta	+
59	IL1RN	Interleukin 1 receptor antagonist	+
60	IL2	Interleukin 2	+
61	IL3	Interleukin 3 (colony-stimulating factor, multiple)	−
62	IL4	Interleukin 4	−
63	IL5	Interleukin 5 (colony-stimulating factor, eosinophil)	−
64	IL6	Interleukin 6 (interferon, beta 2)	+
65	IL7	Interleukin 7	+
66	IL8	Interleukin 8	+
67	IL9	Interleukin 9	−
68	IL10	Interleukin 10	−
69	IL11	Interleukin 11	+
70	IL12A	Interleukin 12A	+
71	IL12B	Interleukin 12B	−
72	IL13	Interleukin 13	−
73	IL15	Interleukin 15	+
74	IL16	Interleukin 16	−
75	IL17A	Interleukin 17A	−
76	IL17F	Interleukin 17F	−
77	IL18	Interleukin 18 (interferon-gamma-inducing factor)	+
78	IL21	Interleukin 21	−
79	IL23A	Interleukin 23, alpha subunit p19	+
80	IL22	Interleukin 22	−
81	IL24	Interleukin 24	−
82	IL27	Interleukin 27	+
Cytokines			
83	ADIPOQ	Adiponectin, C1Q and collagen domain containing	−
84	IFNG	Interferon, gamma	−
